# Phytofabricated gold nanoparticles as modulators of salt stress responses in spinach: implications for redox homeostasis, biochemical and physiological adaptation

**DOI:** 10.3389/fpls.2024.1408642

**Published:** 2024-06-18

**Authors:** Mohammad Amir, Abdul Raheem, Pushpanjali Yadav, Vijay Kumar, Rajesh Kumar Tewari, Syed Uzma Jalil, Mohammad Danish, Mohammad Israil Ansari

**Affiliations:** ^1^ Department of Botany, University of Lucknow, Lucknow, India; ^2^ Amity Institutes of Biotechnology, Amity University, Lucknow, India; ^3^ Botany section, Maulana Azad National Urdu University, Hydrabad, India

**Keywords:** antioxidants, AsA-GSH pathway, gold nanoparticles, plant growth, redox homeostasis, salt stress

## Abstract

**Introduction:**

The utilization of plant material for synthesizing nanoparticles effectively triggers physiological and biochemical responses in plants to combat abiotic stresses. Salt stress, particularly caused by NaCl, significantly affects plant morphology and physiology, leading to reduced crop yields. Understanding the mechanisms of salt tolerance is crucial for maintaining crop productivity.

**Methods:**

In this study, we examined the effects of 150 μM spinach-assisted gold nanoparticles (S-AuNPs) on various parameters related to seed germination, growth attributes, photosynthetic pigments, stomatal traits, ion concentrations, stress markers, antioxidants, metabolites, and nutritional contents of spinach plants irrigated with 50 mM NaCl.

**Results:**

Results showed that S-AuNPs enhanced chlorophyll levels, leading to improved light absorption, increased photosynthates production, higher sugar content, and stimulated plant growth under NaCl stress. Stomatal traits were improved, and partially closed stomata were reopened with S-AuNPs treatment, possibly due to K^+^/Na^+^ modulation, resulting in enhanced relative water content and stomatal conductance. ABA content decreased under S-AuNPs application, possibly due to K^+^ ion accumulation. S-AuNPs supplementation increased proline and flavonoid contents while reducing ROS accumulation and lipid peroxidation via activation of both non-enzymatic and enzymatic antioxidants. S-AuNPs also regulated the ionic ratio of K^+^/Na^+^, leading to decreased Na^+^ accumulation and increased levels of essential ions in spinach plants under NaCl irrigation.

**Discussion:**

Overall, these findings suggest that S-AuNPs significantly contribute to salt stress endurance in spinach plants by modulating various physiological attributes.

## Graphical Abstract

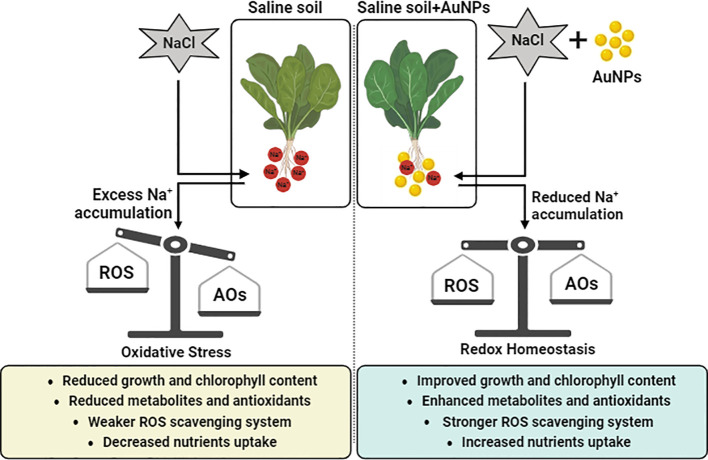

## 1 Introduction

Salt (NaCl) stress poses a significant threat to agricultural productivity globally, particularly in arid and semiarid regions, impacting approximately one-third of agricultural lands, including irrigated areas (Jalil and Ansari, 2020; Mehra and Bennett, 2022). It is projected that about fifty percent of the world’s agricultural land will be saline till 2050 (Hossain, 2019). Salinity disrupts every growth stages of plants, from seed germination to fruiting, leading to decreased biomass and crop yield (Zhao et al., 2021).

Salt stress is known to adversely affect crucial biochemical and physiological processes in plants, disrupting water channel, ionic equilibrium (Na^+^/K^+^), and cellular stability (Agami, 2013; Mohamed et al., 2020). In this situation, plants experienced ions-dependent growth, leading to partial stomatal closure (Rajendran et al., 2009). This also led to deceleration in metabolic process, resulting in premature senescence and eventual plant death (Yang and Guo, 2018; Gong, 2021; Zhao et al., 2021). Beside this, photosynthates production, protein synthesis and lipid metabolism, are also affected under salt stress within a plant (Munns and Tester, 2008; Isayenkov, 2012) and consequently elicits reactive oxygen species (ROS) machinery which trigger oxidative strain and sub-cellular toxicity in plant tissues (Kesawat et al., 2023).

Plants have developed strategies to minimize oxidative damage from salt stress by regulating antioxidant defense systems, both enzymatic and non-enzymatic, to protect cellular machinery from excess ROS production (Agami, 2013; Abdelaziz et al., 2018). Furthermore, the biosynthesis of osmo-protectant (proline) and compatible solute (sugar) are regarded as playing an adaptive role in facilitating osmotic adjustment and safeguarding the sub-cellular structures in plants under salt stress.

In this context, the application of nanotechnology, specifically employing metals nanoparticles (NPs) offers a remedy for reducing the generation of ROS (Jalil and Ansari, 2021; Manaf et al., 2021; Kumari et al., 2022). Many studies show that nanoparticles (NPs) can mitigate salt stress in various plant species by regulating hormones, boosting antioxidant enzymes, balancing ions, controlling AsA-GSH cycle enzymes, and impacting gene expression (Al-Khayri et al., 2021; I. Khan et al., 2020; Zafar et al., 2024). In specific, gold nanoparticles (AuNPs) has been recognized as the most considered metallic NPs, owing to their stability, resistance to oxidation, antimicrobial characteristics, and low toxicity (Balkrishna et al., 2023; P. V. Noruzi, 2015; Kumar et al., 2019). Additionally, AuNPs offer synchronized nutrient release, enhancing plant uptake efficiency; therefore, the promotion of AuNPs based nano-formulations has increased in recent times (Solanki et al., 2015). Several recently conducted research illustrated that the AuNPs mitigated ROS accumulation through activation of the antioxidant machinery i.e., AuNPs on *Nicotiana tabacum* (Jalil et al., 2019), *Spinacia oleracea* (Amir et al., 2024), *Arabidopsis thaliana* (Kumar et al., 2013), AuNPs on *Lavandula angustifolia* (Jadczak et al., 2020). In summary, these researchers have determined that AuNPs might trigger the ROS detoxification system through up regulating the antioxidant enzymes signaling genes (Manaf et al., 2021; Zia-ur-Rehman et al., 2023). Hence, the incorporation of AuNPs has enhanced the potential for crop cultivation in saline soils, leading to improved crops productivity.

*Spinacia oleracea* (Spinach) is a significant annual leafy vegetable known for its abundant vitamins and minerals, along with adequate fibers and low calories (Lakshmi et al., 2017). Recently, there has been a growing demand for spinach as a source of iron. However, due to erratic climate, including salt stress, the yield of spinach has drastically reduced (Ors and Suarez, 2016). Considering aforementioned roles of AuNPs, the presented study investigates how AuNPs supplementation mitigates the ROS production and modulates antioxidants defense systems, subsequently influencing spinach growth markers, physiology, and yield under salinity. Furthermore, our hypothesis posits that AuNPs trigger the activation of AsA-GSH pathway, proline and sugar metabolism, under salt stress conditions. Additionally, our research seeks to uncover the mechanisms through which AuNPs alleviates the adverse effects of salt stress, facilitating redox homeostasis and regulating stomatal traits to improve plant growth.

## 2 Materials and methods

### 2.1 Fabrication of AuNPs and their characterization

The fabrication of gold nanoparticles (AuNPs) using spinach leaf extract was carried out following our previously established protocol as detailed in Amir et al. (2024). After synthesis, size and morphology were analyzed using transmission electron microscope (TEM) [Tecnai G2 Spirit BioTwin, FEI].

### 2.2 Plant material and growth environments

*Spinacia oleracea* L. cv. allgreen (Spinach) seeds obtained from the National Seed Corporation, Lucknow, were subjected to surface sterilization using a 0.01% solution of mercuric chloride (HgCl_2_). These seeds were then cultivated in 13×11 cm pots filled with a re-formed soil consisting of 70% sand, 20% clay, and 10% peat by dry weight. The cultivation took place in a growth chamber located at the Botany Department, University of Lucknow. Growth chamber was maintained with temperature of 24°C ± 3, at a photoperiod cycle of 16/8 hours light/dark, light intensity of 300 nmol m^-2^s^-1^ and 60% humidity.

### 2.3 Salt treatments and S-AuNPs application

During the experiment, the efficacy of 150 μM S-AuNPs was evaluated for mitigating the adverse effects of salt stress induced by sodium chloride (NaCl) in spinach. The concentration of NaCl was selected based on preliminary screening experiments (data not shown). Four treatment sets were established: the first set served as the control and received no treatment; the second set was subjected to salt stress with 50 mM NaCl; the third set was treated with 150 μM S-AuNPs according to findings from our previously published study (Amir et al., 2024); and the fourth set underwent salt stress (50 mM NaCl) followed by treatment with S-AuNPs (150 μM). The required concentrations of NaCl and S-AuNPs were dissolved in double-distilled water (DDW) and applied to the soil at 15 days after sowing (DAS). Sampling was performed at 30, 45, and 60 DAS to evaluate a range of morphological, physiological, and biochemical attributes. The experimental setup involved all treatments being organized in a completely randomized manner with three replicates (n = 3) for each treatment.

### 2.4 Seed germination parameters

Randomly selected spinach seeds were surface sterilized using a 0.01% HgCl_2_ solution for 15 seconds, followed by rinsing four times with DDW to eliminate any residual sterilizing agent. Subsequently, 50 seeds per treatment group were placed in four distinct glass petri plates containing DDW, a 50 mM NaCl solution, a 150 μM S-AuNPs solution, and a NaCl + S-AuNPs solution respectively. These plates were then incubated in a dark chamber (24°C ± 2) for initial 5 days, followed by an additional 2 days under a natural photoperiod at room temperature (25°C ± 3). Three separate replication sets were prepared to assess variability. Germination indices were determined using formulas from previously established protocol (Thomson and El-Kassaby, 1993; Fetouh and Hassan, 2014).

### 2.5 Plant growth analysis

Shoot and root length were measured with a metric scale. The shoot and root fresh weight (fw) of both treated and control plants were assessed using an electronic balance (PGB220, WENSAR). Subsequently, the shoot and root samples were dried out at 48°C for 48 hours and then dehydrated at 70°C for additional 72 hrs to observed their dry mass (Taïbi et al., 2016). Leaf numbers were quantified by manual leaf counting, while leaf area was taken using a leaf area meter (LAM 21, Systronics), as described by Arora et al. (2012).

### 2.6 Photosynthetic pigments

Fresh leaf weighing 0.5 g were homogenized with 80% (v/v) chilled acetone to extract chlorophyll and carotenoid content followed by centrifugation (REMI Neya-16R, India)) at 10,000 rpm for 10 minutes (min). For chlorophyll, the optical density (O.D.) of the samples was measured at wavelengths of 663 and 645 nm with a UV-Vis spectrophotometer [Shimadzu UV-1601, Tokyo, Japan] following the protocol of Arnon (1949). Further, carotenoid content was calculated by taking the sample O.D. at 480 and 510 nm, as outlined by Maclachlan and Zalik (1963).

### 2.7 Measurement of stomatal dynamics and abscisic acid estimation

Stomatal traits, such as stomatal frequency, were measured according to protocol outlined by Salisbury (1928) and stomatal index was calculated based on the formulation described by Xu and Zhou (2008). Furthermore, the transpiration rate was observed before noontime, using Ganang’s Potometer (Singh et al., 2014). The abscisic acid (ABA) concentration was determined by following the method of Hung and Kao (2003).

### 2.8 Scanning electron microscopic imaging

Stomata morphology was investigated with a protocol outlined by Faizan et al. (2021). Fresh leaf specimens were immediately preserved with methanol, followed by immersion in a series of diluted ethanol solution. Further, the dehydrated sample was coated with gold and pore length and width was observed with a scanning electron microscope (SEM) [JEOL SM-6510, Tokyo, Japan].

### 2.9 Oxidative stress markers, electrolyte leakage and lipoxygenase activity

Lipid peroxidation (MDA) content was assessed using the method outlined by Heath and Packer (1968). Fresh leaf specimen weighing 0.5 g was extracted with 0.1% trichloroacetic acid (TCA) and then centrifuged at 12000 rpm at 4°C for 15 min. Resulting supernatant was mixed with 0.5% thiobarbituric acid made in 20% TCA. The mixture was then heated at 95°C for 30 minutes and subsequently cooled in an ice bath. After homogenizing the reaction mixture at 1000×g for 15 min. at 4°C, the absorbance of the supernatant was measured at 532 nm.

The hydrogen peroxide (H_2_O_2_) content was determined using the method described by Patterson et al (1984). A fresh leaf sample weighing 500 mg was extracted with acetone and then centrifuged at 8000 rpm for 15 min. The resulting supernatant was mixed with a solution containing 20% titanium chloride, concentrated HCl and 17 M ammonia. The obtained precipitate was washed multiple times with acetone, after which 2N H_2_SO_4_ was added to it. The absorbance was recorded at 410 nm, and H_2_O_2_ content was calculated from a standard curve prepared using H_2_O_2_, expressed as μmol g^−1^ fw.

The content of Superoxide anion (O_2_^˙−^) was determined according to the procedure described by López et al. (2009). The O_2_^˙−^ was quantified using a sodium nitrite standard curve and were represented as μmole g^–1^ fw.

Electrolyte leakage (EL) was determined using the electrical conductivity method as described in our previous study (Jalil et al., 2017). Activity of lipoxygenase (LOX, EC 1.3.11.12) was assessed following the method outlined by Surrey (1964).

### 2.10 In-situ localization of H_2_O_2_ in leaf

Dichlorodihydrofluorescein diacetate (H_2_DCF-DA) was employed for the qualitative assessment of H_2_O_2_ accumulation (Kumar et al., 2022). For this purpose leaf peels of different treatment groups were immersed in buffer (5 mM MES-KOH, pH 5.8, 5 mM CaCl_2,_ 0.5 mM sorbitol) containing 10 mM H_2_DCF-DA, incubated at RT in dark condition for 30 min, and then washed three times with 5 mM MES buffer (pH 5.8) and were mounted slides using same buffer. Finally, the fluorescence image was observed under a fluorescence microscope (Magnus-MLX1 plus) using FITC filter; excitation and emission of 490 and 515 nm respectively.

### 2.11 Proline estimation

The Proline content was estimated following the protocol established by Bates et al. (1973). Fresh leaf sample weighing 0.1 g was crushed with 5 mL of 3% aqueous sulphosalicylic acid and then centrifuged at 10,000 rpm for 15 min. An aliquot of 1 mL was mixed with acidic ninhydrin and glacial acetic acid, incubated at 100°C for 10 min, and subsequently cooled in an ice bath. Further, the mixture was extracted with 4 mL toluene, vortexed for 20 s, and cooled. The absorbance at 520 nm was recorded, and the free proline content was determined from a standard curve, expressed as μmol g^-1^ fw.

### 2.12 Non–enzymatic antioxidant markers and sugar contents

Ethanol extracts from leaf were prepared to assess non-enzymatic antioxidants and sugar content. Leaf sample weighing 0.5 g was extracted with 10 mL of 80% ethanol and then filtered through Whatman No. 41 filter paper. The residue was re-homogenized with ethanol, and polled together to make a final volume of 20 mL. From the prepared extracts, determination of total sugars were assessed following the method outlined by DuBois et al. (1956), flavonoids were assessed as per Pękal and Pyrzynska (2014) and ascorbate (AsA) was determined from the according to Azuma et al. (1999).

Glutathione (GSH) content was estimated by using protocol as defined by Anderson (1985). A leaf samples weighing 0.5 g were homogenized with an extraction buffer containing 5% (w/v) sulphosalicylic acid and then centrifuged at 10,000×g for 10 min. Subsequently, 0.5 mL of 100 mM PBS and 40 mL of 5, 5′ -dithiobis-2-nitrobenzoic acid were added to 0.5 mL of the supernatant, and the absorbance was recorded at 412 nm.

### 2.13 Antioxidant enzymes activities determination

The antioxidant enzymes extraction procedure was conducted at 4°C following the procedure outlined by Dwivedi et al. (2020). Fresh leaf samples weighing 0.5 g were homogenized with 5 mL of 100 mM PBS (pH 7.6), which included 1 mM EDTA.Na_2_, 0.5 mM ascorbate, and 1% Polyvinylpyrrolidone. The homogenates were then centrifuged at 10,000 rpm for 5 min, and the resulting supernatant was utilized as the crude extract for enzymes estimation.

Catalase (CAT, EC 1.11.1.6) activity was determined by measuring the reduction in H_2_O_2_ and monitoring absorbance difference at 240 nm, following the method described by Aebi (1984).

Superoxide dismutase (SOD, EC 1.15.1.1) activity was assessed using the nitroblue-tetrazolium method, as described by Chen and Pan (1996).

Peroxidase (POD, EC1.11.1.7) activity was measured by monitoring the difference in absorbance at 420 nm, reaction mixture consist of pyrogallol, PBS, H_2_O_2_, and enzyme extract, following the method outlined by Chance and Maehly (1955).

Ascorbate peroxidase (APX, EC 1.11.1.11) activity was assessed by observing the reduction in ascorbate and observing the difference in absorbance at 290 nm, according to the method described by Nakano and Asada (1981).

Glutathione peroxidase (GPX, EC 1.11.1.9) activity was calculated by recording the decrease in absorbance at 340 nm caused by the NADPH oxidation at 25°C, following the protocol outlined by Smith and Johnson (1988).

Glutathione reductase (GR, EC 1.8.1.7) activity was measured by observing the decline in absorbance at 412 nm resulting from oxidation of NADPH, according to the method described by Halliwell and Foyer (1978).

### 2.14 Nutrient contents estimation

For nutrient analysis, samples were prepared following the method outlined by Kumar et al. (2014). Leaf samples were washed two times with DDW and immersed in 20 mM EDTA for 5 second. Afterward, the samples washed again with DDW twice to ensure thorough removal of any remaining metal on the plant surface. The washed samples were subsequently dehydrated in an oven at for 105°C for 24 hours. Further, dried samples were digested by using a wet digestion method with a mixture of HNO_3_: HClO_4_ (7:3, V/V) until clear precipitate were achieved. Each sample was then diluted with DDW to a final volume of 20 mL. The concentrations of Sodium (Na), potassium (K), calcium (Ca), iron (Fe), manganese (Mn), and zinc (Zn) in the leaf samples were analyzed with Atomic Absorption Spectrophotometer (AAS) [Agilent 240FS-AA].

### 2.15 Statistical analysis

All Measurements were carried out in a randomized manner across three replicates (n=3) for each treatment group. The data presented are mean values accompanied by their corresponding standard deviation (SD). Statistical analyses, including one-way analysis of variance (ANOVA) and Duncan’s multiple range test (DMRT), were performed to identify the significant difference between treatments at 5% level (P ≤ 0.05) by using SPSS 20.0 software (SPSS, Inc., Chicago, IL, USA). Additionally, principal component analysis (PCA) was conducted to assess the correlation between the studied variable and treatment by using OriginPro 2018 software (Origin-Lab Corporation, Northampton, MA, USA).

## 3 Results

### 3.1 S-AuNPs synthesis and characterization

**Figure 1:**
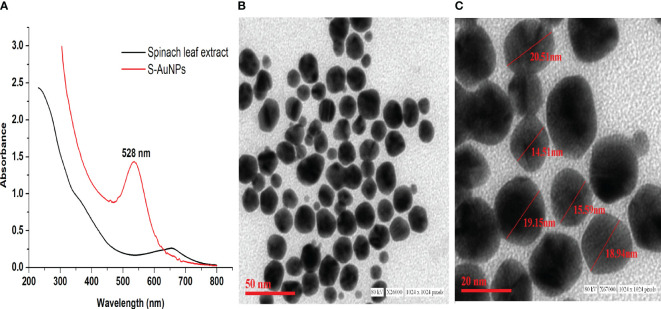
**(A)** Synthesized S-AuNPs UV-Vis spectral graph, **(B)** transmission electron microscope (TEM) micrograph at scale bars of 50 nm, and **(C)** 20 nm.

The synthesis of AuNPs was accomplished using 1 mM chloroaurate (HAuCl_4_) as the gold precursor and 0.33 mg/ml spinach leaf extract as reducing agent. The gold reduction process was visually validated by observing the color transformation of the reaction mixture from pale yellow to ruby red. Subsequent UV-Vis spectral scanning verified the formation of AuNPs, showing an absorption peak at 528 nm corresponding to the AuNPs SPR band (**Figure 1A**). Additionally, TEM was employed to assess the core size, shape, and 2-dimensional morphology of synthesized S-AuNPs. The analysis revealed a spherical shape with monodispersed distribution and core size ranging from 14.5 to 20.5 nm (**Figures 1B, C**).

### 3.2 S-AuNPs mediated alleviation of NaCl-induced changes in seed germination indices

**Table 1** illustrated that spinach seeds treated with NaCl exhibited a negative impact on germination-related parameters. However, when supplemented with S-AuNPs + NaCl, increases in GP by 20.4%, GE by 7.6%, and MDG by 6.4% were recorded against NaCl treatment.

**Table 1:** Data presented here shows the germination parameters of *S. oleracea* seed grown in petriplates individually with control, 50 mM NaCl, 150 µM AuNPs and 50 mM NaCl + 150 µM AuNPs.

Parameters	Control	NaCl	AuNPs	NaCl + AuNPs
Germination Percentage (%)	87.50 ± 5.59b	62.50 ± 5.59d	91.67 ± 3.23a	70.83 ± 6.45c
Germination Energy	0.88 ± 0.06ab	0.63 ± 0.06d	0.92 ± 0.03a	0.71 ± 0.06b
Mean Daily Germination	14.58 ± 0.93a	10.42 ± 0.93b	15.28 ± 0.54a	11.83 ± 1.08ab

Each value represents mean ± SD of three replicates. Means followed by the same letter (s) do not differ by DMRT test at 5% probability level (p ≤ 0.05).

### 3.3 S-AuNPs mediated amelioration of NaCl-induced changes in growth and biomass

**Figure 2:**
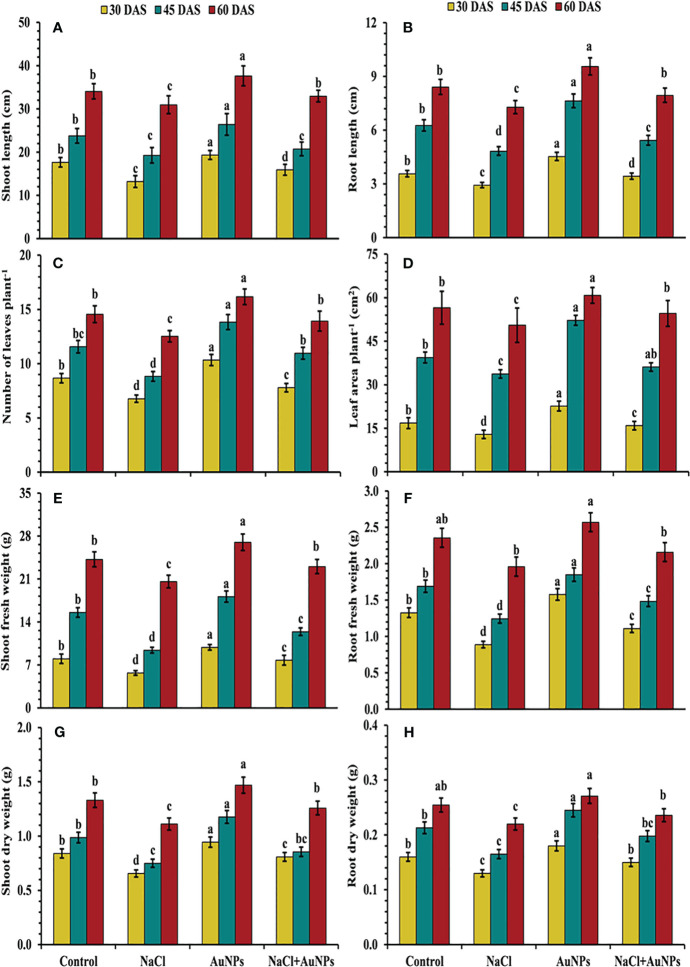
**(A)** Shoot length, **(B)** root length, **(C)** number of leaves per plant, **(D)** leaf area, **(E)** shoot fresh weight, **(F)** root fresh weight, **(G)** shoot dry weight, and **(H)** root dry weight in *S. oleracea* plant grown individually with control, 50 mM NaCl, 150 µM S-AuNPs and 50 mM NaCl + 150 µM S-AuNPs at 30, 45, and 60 DAS. Each bar represents mean ± SE of three replicates. Means followed by the same letter (s) do not differ by DMRT test at 5% probability level (p ≤ 0.05).

From **Figure 2**, it is evident that plants exposed to salt stress exhibited reduced growth characteristics, including plant length, leaf number, leaf area and biomass as compared to control plants. However, these parameters were improved by the S-AuNPs application under salt stressed conditions. Shoot lengths were increased by 20.4%, 7.6%, and 6.4%, at 30, 45, and 60 DAS, respectively when supplemented with S-AuNPs + NaCl compared to NaCl treated plants (**Figure 2A**). A similar trend was observed in root length, with increases of 17.4%, 12.4%, and 9.0% at 30, 45, and 60 DAS, respectively (**Figure 2B**). Additionally, leaf number increased by 15.0%, 24.1%, and 11.2%, and leaf area by 23.1%, 7.0%, and 8.0%, respectively, at 30, 45, and 60 DAS against NaCl treated plants (**Figures 2C, D**). Shoot and root fresh mass increased by 36.3%, 31.8%, and 11.9%, and 25.0%, 19.3%, and 10.2%, at 30, 45, and 60 DAS respectively (**Figures 2E, F**). The shoot and root dry mass was also increased in all plants treated with S-AuNPs, with respective increases of 23.3%, 14.0%, and 13.0% for shoot dry mass and 15.3%, 15.0%, and 7.4% for root dry mass, over the salt stressed plants at 30, 45, and 60 DAS (**Figures 2G, H**).

### 3.4 S-AuNPs mediated augmentation of photosynthetic pigments

**Figure 3:**
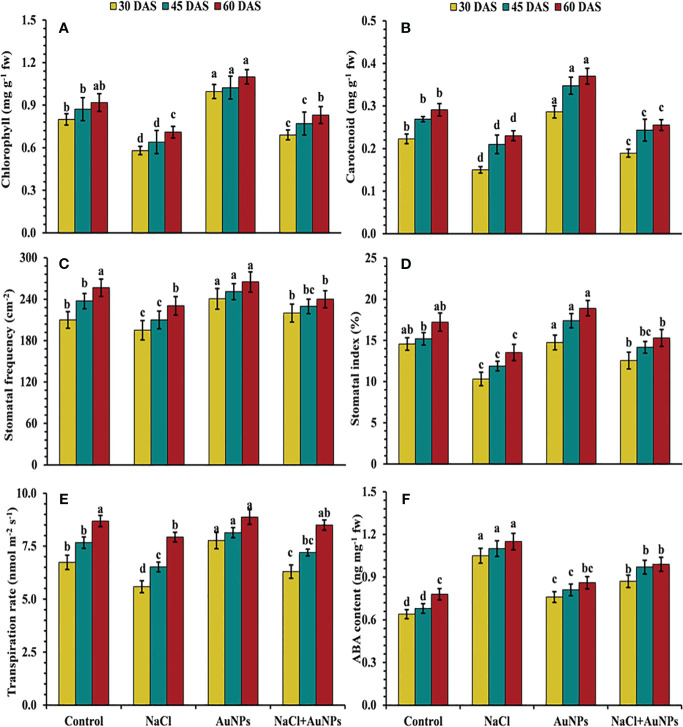
**(A)** Chlorophyll content, **(B)** carotenoid content, **(C)** stomatal frequency, **(D)** stomatal index, **(E)** transpiration rate, and **(F)** abscisic acid content in *S. oleracea* plant grown individually with control, 50 mM NaCl, 150 µM S-AuNPs and 50 mM NaCl + 150 µM S-AuNPs at 30, 45, and 60 DAS. Each bar represents mean ± SE of three replicates. Means followed by the same letter (s) do not differ by DMRT test at 5% probability level (p ≤ 0.05).

From **Figure 3**, it is clear that the chlorophyll contents were decreased by 27.4%, 26.6%, and 22.8% respectively at 30, 45, and 60 DAS under salt stress compared to control plants. However, when supplemented with S-AuNPs with NaCl, chlorophyll content were found to be increased by 19.0%, 20.3%, and 16.9%, respectively compared to NaCl treated plants. In contrast to chlorophyll content, carotenoid content was also improved by 26.1%, 15.8%, and 10.9%, respectively at 30, 45, and 60 DAS when supplemented with S-AuNPs + NaCl in comparison to salt treated plants (**Figure 3B**).

### 3.5 S-AuNPs mediated improvement of stomatal traits and abscisic acid modulation

**Figure 3** illustrates that plants treated with NaCl experienced reduced stomatal traits (stomatal frequency, index, aperture and transpiration rate) as compared to control plants. However, applying S-AuNPs to salt affected spinach plants led to an increase in stomatal frequency by 12.8%, 9.3%, and 8.7%, and stomatal index by 21.7%, 19.8%, and 14.1%, respectively at 30, 45, and 60 DAS as compared to salt treated plants (**Figures 3C, D**). Similarly, with the combined treatment of AuNPs and NaCl, transpiration rate increased by 12.7%, 10.4%, and 7.4%, respectively at 30, 45, and 60 DAS in comparison to salt-treated plants (**Figure 3E**). In response to the salt stress, the ABA level was increased by 64.0%, 51.7%, and 47.4% against control. However, the combined treatment of S-AuNPs and NaCl reduced the ABA level by 17.1%, 13.7%, and 13.9%, respectively at 30, 45, 60 DAS compared to NaCl treated plants (**Figure 3F**).

### 3.6 S-AuNPs mediated regulation of stomatal aperture

**Figure 4:**
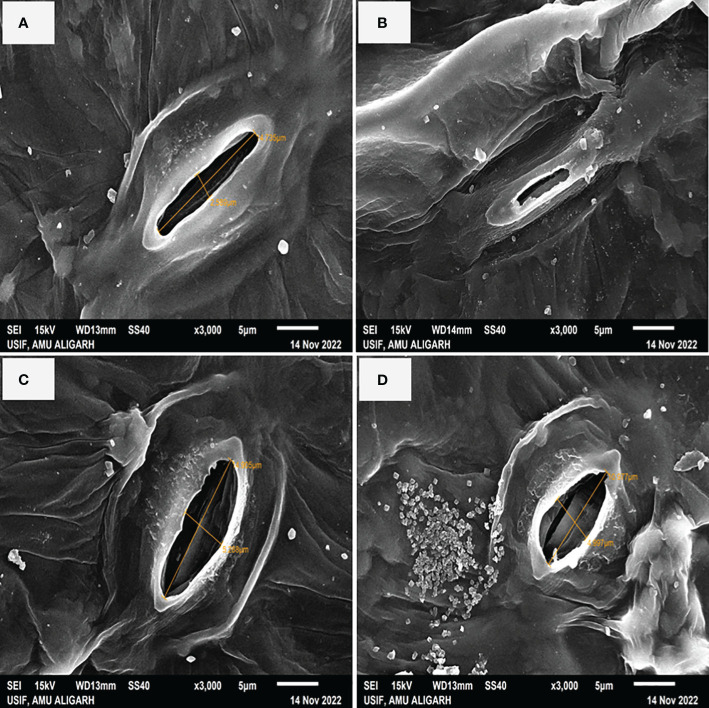
Scanning electron microscope (SEM): response of stomatal aperture; at 30 days old S. oleracea leaves treated with **(A)** control, **(B)** 50 mM NaCl, **(C)** 150 µM S-AuNPs, and **(D)** 50 mM NaCl + 150 µM S-AuNPs at × 3000 magnification.

In **Figure 4** it is shown that plants treated with NaCl had partially closed stomata with reduced aperture size by 34.79% as compared to control plants (**Figures 4A, B**). Conversely, the application of S-AuNPs alone showed a wider aperture compared to control plants (**Figure 4C**). However, when S-AuNPs were applied to salt affected spinach plants, the aperture size increased by 20% as compared to salt-treated plants but remained less than that of control plants (**Figure 4D**).

### 3.7 S-AuNPs mediated down regulation of oxidative stress markers

**Figure 5:**
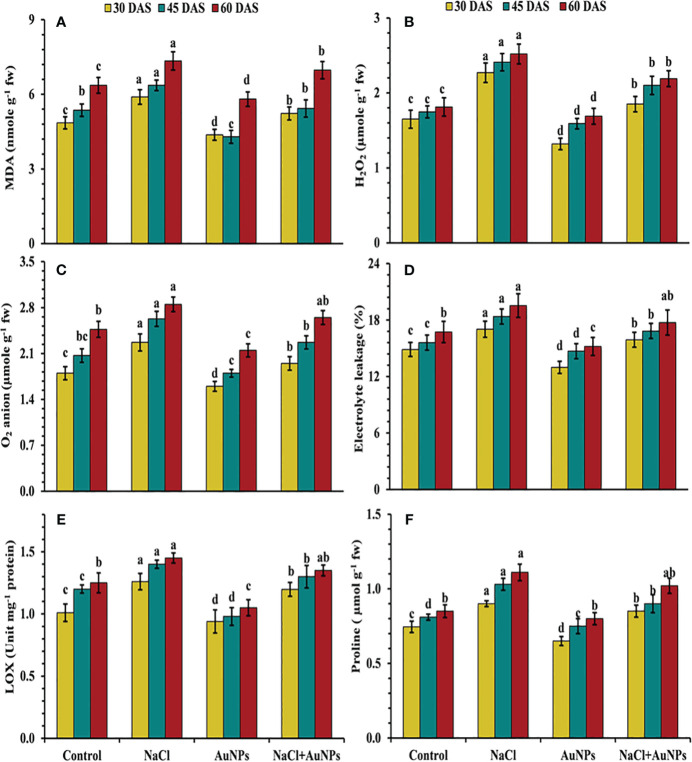
**(A)** MDA content, **(B)** H_2_O_2_ level, **(C)** superoxide anion content, **(D)** ion leakage, **(E)** lox activity, and **(F)** proline content in *S. oleracea* plant grown individually with control, 50 mM NaCl, 150 µM S-AuNPs and 50 mM NaCl + 150 µM S-AuNPs at 30, 45, and 60 DAS. Each bar represents mean ± SE of three replicates. Means followed by the same letter (s) do not differ by DMRT test at 5% probability level (p ≤ 0.05).

From **Figure 5** it is indicated, that the plants exposed to salt stress exhibited increased production of oxidative stress markers as compared to control plants. However, under combined application of S-AuNP + NaCl, leaf MDA content was decreased by 11.2%, 14.7%, and 5.0%, while H_2_O_2_ was decreased by 18.5%, 12.9%, and 13.0% respectively at 30, 45, and 60 DAS in comparison to NaCl treated plants (**Figures 5A, B**). A similar trend was observed in superoxide anion content, with decrease of about 14.1%, 13.7%, and 7.0%, respectively with application of S-AuNPs in salt stressed plant over to NaCl treated plants (**Figure 5C**) Beside this, the electrolyte leakage was decreased by 6.6%, 8.4%, and 9.2%, lipoxygenase content was decreased by 4.9%, 10.3%, and 3.6% respectively at 30, 45, and 60 DAS against NaCl treated plants (**Figures 5D, E**).

### 3.8 In-situ localization of H_2_O_2_

**Figure 6:**
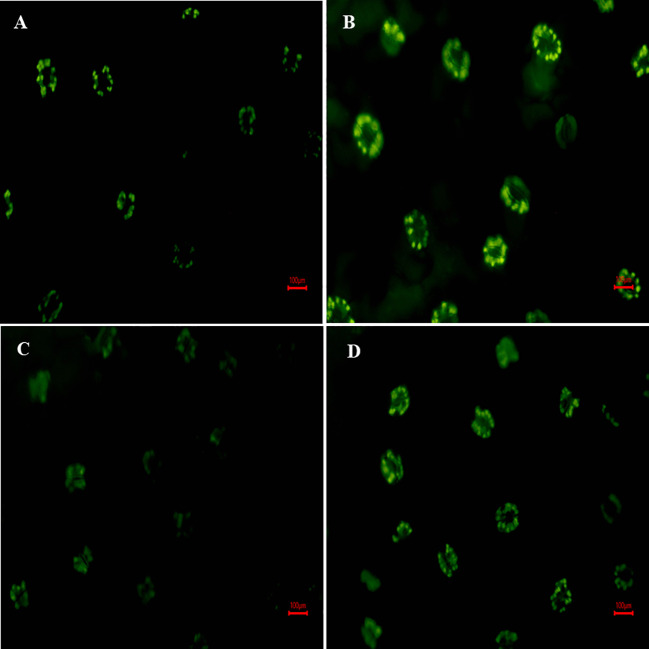
Fluorescence microscopy response of *in-situ* localization of H_2_O_2_; at 30 days old *S. oleracea* leaves treated with **(A)** control, **(B)** 50 mM NaCl, **(C)** 150 µM S-AuNPs, and **(D)** 50 mM NaCl + 150 µM S-AuNPs at 40X.

**Figure 6** depicted that, fluorescence microscopy images of H_2_DCF-DA fluorescence were used to characterize the formation of H_2_O_2_ in leaves, where fluorescence intensity is directly proportional to H_2_O_2_ accumulation. Results illustrate that under salt exposure, H_2_DCF fluorescence intensity was increased (brighter green color) against control plants (**Figures 6A, B**). However, the combined treatment of S-AuNPs and NaCl reduced the fluorescence intensity against NaCl treated plants (**Figures 6C, D**), as observed by appearance of diminished green color.

### 3.9 S-AuNPs mediated modulation of osmolyte

**Figure 5F** illustrate that upon salt exposure proline content was increased by 20.0%, 27.16% and 30.59% at 30, 45, and 60 DAS, respectively as compared to control. However, combined treatment of S-AuNPs and NaCl reduced the proline content by 5.7%, 12.6% and 8.11% respectively at 30, 45, and 60 DAS compared to salt treated spinach plant.

### 3.10 S-AuNPs mediated improvement of metabolites and non-enzymatic antioxidants

**Figure 7:**
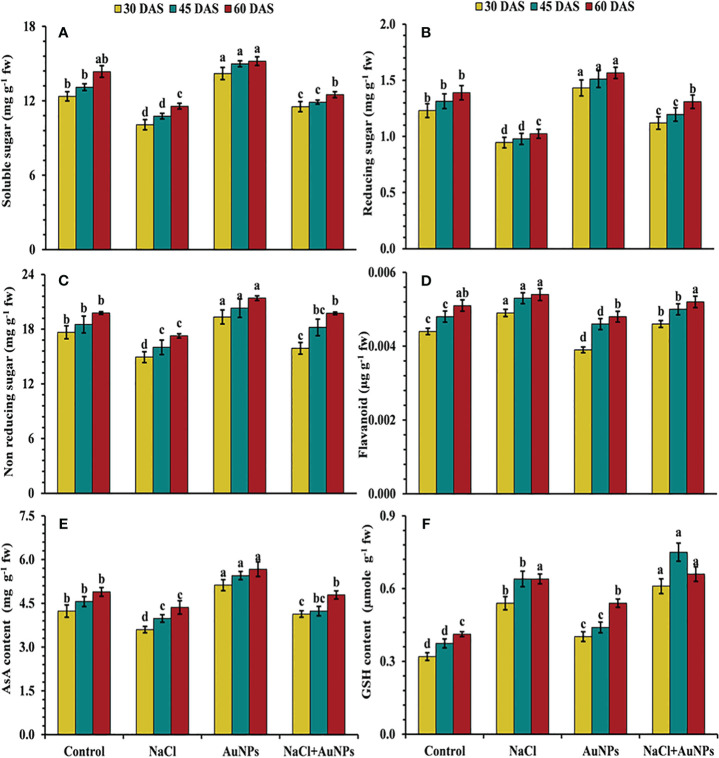
**(A)** Total soluble sugar, **(B)** reducing sugar, **(C)** non reducing sugar, **(D)** flavonoid content, **(E)** ascorbate, and **(F)** glutathione content in *S. oleracea* plant grown individually with control, 50 mM NaCl, 150 µM S-AuNPs and 50 mM NaCl + 150 µM S-AuNPs at 30, 45, and 60 DAS. Each bar represents mean ± SE of three replicates. Means followed by the same letter (s) do not differ by DMRT test at 5% probability level (p ≤ 0.05).

From **Figure 7** it is marked that the level of soluble sugar, including reducing and non-reducing sugars were decreased under salinity as compared to control plants. Total soluble sugar was found to be increased by 14.5%, 10.4% and 8.4% at 30, 45, and 60 DAS, respectively, when treated with S-AuNPs with NaCl as compared to salt treated plants (**Figure 7A**). A similar trend to that of total soluble sugar was observed in reducing and non-reducing sugar (**Figures 7B, C**). Non enzymatic antioxidant flavonoid, AsA and GSH content were also affected under salt stressed plant. Flavonoid content were found to be decreased by 6.1%, 5.7% and 9.8% respectively at 30, 45, and 60 DAS, when supplemented with S-AuNPs + NaCl against NaCl treated plants (**Figure 7D**). Ascorbate content were increased by 14.8%, 6.3% and 9.8%, reduced glutathione content increased enormously under salt stress but were respective increase of 13.0%, 17.2% and 3.4% respectively under co-application of S-AuNPs and NaCl as compared to NaCl treated spinach plants (**Figures 7E, F**).

### 3.11 S-AuNPs mediated regulation of enzymatic activity

**Figure 8:**
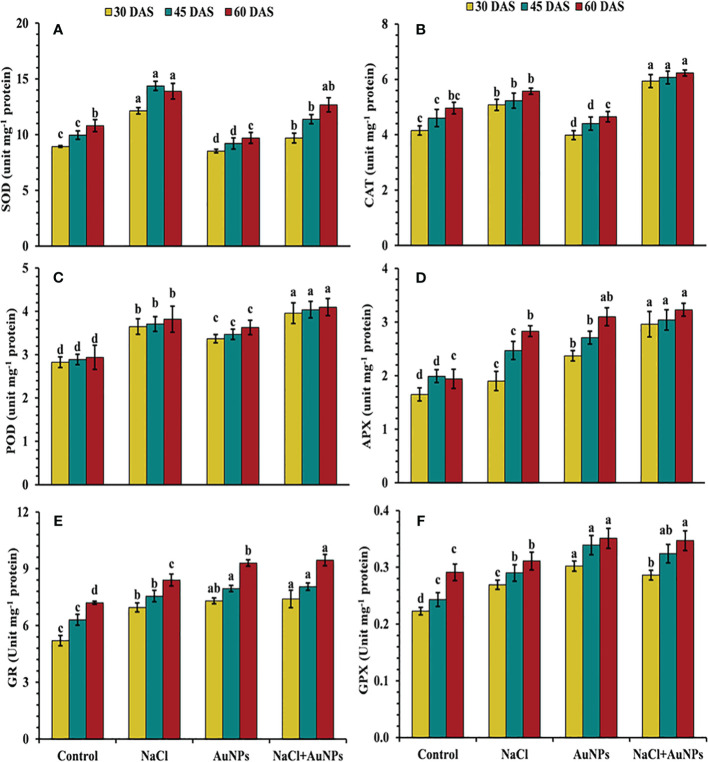
**(A)** Superoxide dismutase, **(B)** catalase, **(C)** peroxidase, **(D)** ascorbate peroxidase, **(E)** glutathione reductase, and **(F)** glutathione peroxidase content in *S. oleracea* plant grown individually with control, 50 mM NaCl, 150 µM S-AuNPs, and 50 mM NaCl + 150 µM S-AuNPs at 30, 45, and 60 DAS. Each bar represents mean ± SE of three replicates. Means followed by the same letter (s) do not differ by DMRT test at 5% probability level (p ≤ 0.05).

**Figure 8** results illustrate that increased antioxidant enzyme activities were observed in salt-stressed spinach plants compared to the control. However, the combined treatment of S-AuNPs with NaCl, further improved these enzymes activities as compared to salt-stressed plants. Specifically, CAT activity increased by 16.0%, 41.3%, and 26.7%, POD activity by 4.2%, 7.0%, and 16.4%, and APX activity by 61.0%, 23.0%, and 14.3%, respectively, at 30, 45, and 60 DAS, compared to respective salt-treated plants (**Figures 8B–D**). Conversely, under the combined treatment of S-AuNPs and NaCl, SOD activity decreased by 20.3%, 38.8%, and 38.3%, respectively, at 30, 45, and 60 DAS, compared to salt-stressed plants (**Figure 8A**). Furthermore, the activity of GR and GPX increased by 6.5%, 6.7%, and 12.5%, and 6.31%, 11.7%, and 11.5%, respectively, at 30, 45, and 60 DAS, under S-AuNPs and NaCl treatment compared to salt-stressed spinach plants (**Figures 8E, F**).

### 3.12 S-AuNPs mediated improved nutrient uptake

It is marked from **Table 2** that the plants treated with NaCl affected the cationic mineral profiles of spinach. The Na^+^ content and Na^+^/K^+^ ratio was increased by 58.6% and 122% respectively, under NaCl treatment as compared to control. However, they decreased by 23.9% and 28.8% respectively, under S-AuNPs + NaCl supplementation as compared to NaCl stressed plants. Similarly, Na^+^/K^+^ were also surge under salt stress. In contrast, the contents of K^+^, Fe^+2^, Ca^+2^ and Zn^+2^ decreased under salt stress against control. Conversely, combined treatment of S-AuNPs and NaCl increased the K^+^, Ca^+2^, Fe^+2^, Mn^+2^, and Zn^+2^ content by 24.3%, 9.9%, 18.3%, 20.6%, and 29.8% respectively at 45 DAS as compared to salt treated plant.

**Table 2:** Data presented here shows the nutrients (Na, K, Ca, Fe, Mn, and Zn) content (mg g^-1^ DW) of 30 DAS *S. oleracea* leaves grown individually with control, 50 mM NaCl, 150 µM AuNPs and 50 mM NaCl + 150 µM AuNPs.

Nutrients	Control	NaCl	AuNPs	NaCl + AuNPs
Na	13.43 ± 0.91b	25.30 ± 1.72a	10.47 ± 0.32d	16.20 ± 1.42b
K	24.32 ± 1.27b	17.32 ± 0.70d	31.47 ± 1.39a	21.53 ± 1.34c
Ca	8.67 ± 0.35b	6.67 ± 0.28c	12.00 ± 0.90a	7.33 ± 0.23bc
Fe	19.40 ± 0.47b	14.17 ± 0.39d	25.73 ± 0.42a	16.77 ± 0.87c
Mn	9.27 ± 1.08ab	7.17 ± 1.14c	11.53 ± 1.00a	8.65 ± 1.11b
Zn	5.63 ± 0.50b	4.57 ± 0.35c	6.67 ± 0.35da	5.93 ± 0.61b

Each value represents mean ± SD of three replicates. Means followed by the same letter (s) do not differ by DMRT test at 5% probability level (p ≤ 0.05).

### 3.13 Correlation analysis

**Figure 9:**
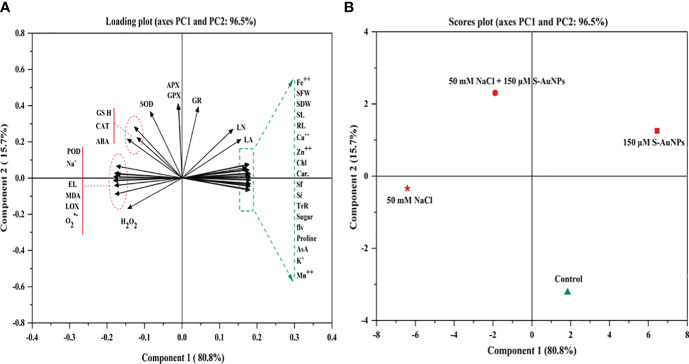
PCA generated variables loading plot **(A)**, and score plot **(B)** of different treatments in *S. oleracea.* Different abbreviations used in the figure are as follows; SL, shoot length; RL, root length; LA, leaf area; NL, number of leaves; SFW, shoot fresh weight; SDW, shoot dry weight; Chl, chlorophyll; Car, carotenoid; Sf, stomatal frequency; Si, stomatal index; TrR, transpiration rate; AsA, ascorbate; GSH, glutathione; ABA, abscisic acid; Pro, proline; MDA, malondialdehyde; H_2_O_2_, hydrogen peroxide; O_2_^˙−^,superoxide anion; EL, electrolyte leakage; LOX, lipoxygenase; POD, peroxidase; APX, ascorbate peroxidase; CAT, catalase; SOD, superoxide dismutase; GR, glutathione reductase; and GPX, glutathione peroxidase.

PCA was utilized to investigate the inter-relationships among the variables under study (**Figure 9**). The loading and score plot revealed significant variations among the variables and treatment groups; with PC1 contributing 80.8% of the variation and PC2 contributes 15.7% variation. Notably, treatments involving S-AuNPs were clustered within the first two components, indicating a substantial impact of S-AuNPs in alleviating the adverse effects on NaCl-treated plants. PC1 predominantly influenced most of the spinach response variables, excluding Na^+^, MDA, LOX, H_2_O_2_, EL and proline, which were primarily represented by PC2. This suggests a correlation and co-variation among the spinach response variables, excluding the mentioned ones. Additionally, in the score plot, S-AuNPs exhibited a significant contribution to PC1 and showed a strong negative correlation with PC2. Furthermore, the positive correlation of S-AuNPs + NaCl with PC1 confirms the beneficial regulatory effect of S-AuNPs in ameliorating the detrimental impact of NaCl stress on spinach plants.

## 4 Discussion

In the last few years, numerous research have been done to unravel the role of noble metal NPs in agriculture as a means to enhance crop production and assure food security (Balkrishna et al., 2023; Paulami et al., 2023; Thabet and Alqudah, 2024). However, phytofabrication of AuNPs, their role in modulating plant responses to abiotic stress, and possible action pathways remain little understood. In this regard, this study provide a series of parametric studies that began with germination traits to observe the crucial roles that phytofabricated AuNPs play in changing plant physiology under salt stress (Ain et al., 2024).

Salinity had a detrimental effect on seed germination percentage, germination energy and average daily germination. However, S-AuNP supplementation significantly improved germination traits under salt stress (**Table 1**). This could be potentially attributed to increased permeability of seed coat (Gopinath et al., 2014), enhancing cells’ ability to absorb entry of di-oxygen and water, which expedites the metabolism (Zheng et al., 2005); resulting in accelerated germination rate, coleoptiles elongation and proper seedlings establishment. Moreover, the improved germination percentage under AuNPs application has also accelerated the shoot growth, biomass and root length (Arora et al., 2012).

Our findings also unveiled that, salinity stress significantly reduced growth biomarkers (**Figure 2**). This might be due to an imbalance in ROS production, membrane damage and changes in antioxidant machinery (Khan et al., 2024); leading to impaired photosynthetic machinery (Karumannil et al., 2023) and stomatal dynamical traits (Wahid et al., 2022). However, following AuNPs treatment, the plants exhibited significant enhancements in various growth attributes. The potential explanation is that NPs applications may reduce Na^+^ translocation to shoot; resulting in increased uptake of essential nutrients reflecting the better growth of plant (Liu and Lal, 2015). Consistent with our findings, the use of AuNPs enhanced plant growth (Arora et al., 2012; Jalil et al., 2019; Kumar et al., 2013) by up-regulating the expression of nitrogen-recycling genes (Wahid et al., 2022). This results in improved photosynthetic efficiency, leading to increased production of photo assimilates and consequently enhancing plant growth (Abdel-Aziz and Rizwan, 2019).

Chlorophyll levels serve as a reliable indicator of photosynthetic activity, particularly under unfavorable conditions (Xu et al., 2008). The data, illustrated significant reduction in chlorophyll and carotenoid contents under salt stress (**Figures 3A, B**). This could be attributed to reduced pigment biosynthesis or degradation (Ma et al., 2017), interaction with different photosynthetic enzymes (Stepien and Johnson, 2009), Suppression of the photosynthetic electron transport chain and closure of stomatal pores (Faizan et al., 2021). Additionally, salt stress often prompts stomatal closure as a plant’s defense mechanism to conserve water, further limiting CO_2_ uptake and exacerbating the decline in photosynthetic pigments. Nevertheless, the addition of S-AuNPs reversed the damage caused by NaCl stress and enhanced pigment content in stressed spinach plants. Our findings are consistent with prior research, which demonstrated that AuNPs treatments have led to elevated chlorophyll levels in mustard (Khatoon et al., 2024), sorghum (Namasivayam and Chitrakala, 2011) and asparagus (An et al., 2008). The mechanism behind this enhancement involves the alleviation of stress-induced inhibition on pigment biosynthesis pathways, possibly through the modulation of enzyme activities and electron transport chain components. By restoring chlorophyll and carotenoid levels, S-AuNPs facilitate greater light absorption by the plants, thereby enhancing photosynthates production. This increase in photosynthates, coupled with improved stomatal conductance resulting from stress amelioration, likely contributes to the observed elevation in sugar content (**Figures 7A–C**) and stimulated plant growth (Urbonaviciute et al., 2006; Muhammad et al., 2023).

Furthermore, during NaCl stress, the effects of S-AuNPs on stomatal properties (stomatal frequency, stomatal index, and transpiration rate) were also investigated. Stomatal aperture size governs both leaf transpiration and gaseous flux; however, stomatal size and density play key roles in gaseous exchange as well as photosynthetic activities (Faraz et al., 2022; Mulyono et al., 2022). Our finding revealed that, salinity caused partial closure of stomata (**Figure 4B**) as well as decreased stomatal frequency and index (**Figures 3C, D)**; leading to decrease stomatal conductance which subsequently reduce the entry of CO_2_. This might be due to K^+^ sequestration, which regulates stomatal traits including frequency, index, and size of aperture (Ahanger and Agarwal, 2017). Moreover, studies have noted that K^+^ and ABA levels impact stomatal opening and closing, governing gas exchange and water movement within plants (Tränkner et al., 2018; Hasan et al., 2021). After S-AuNPs treatment stomatal traits were improved (**Figures 3C–E**) and partially closed stomata were reopened (**Figure 4D**). This might be due to K^+^/Na^+^ modulation, which raised K^+^ levels and decreased Na^+^ accumulation in the leaves under salt-stress, resulted in improved stomatal characteristics and notable rise in relative water content. Our results align with previous research. According to Wahid et al. (2022), combined application of S-AuNPs + NaCl alleviates the stomatal index, frequency, and transpiring area compared to salt treated plants. Faraz et al. (2022), reported that Brassica juncea stomatal density were increased by the administration of CuO-NPs. Additionally, another finding suggest that topical application of NPs to the leaves improved stomatal dynamics and facilitated stomatal opening, thus enhancing photosynthetic activity (Mulyono et al., 2022).

Under salinity stress, ABA concentration significantly increased, representing a self-devised strategy of plants to mimic salt stress by modifying physiological processes such as leaf senescence, closure of stomatal pores and nitrogenous compound accumulation (Vishwakarma et al., 2017). The data presented in the results demonstrate a significant increase in ABA content under NaCl stress (**Figure 3F**). However, S-AuNPs supplementation alleviates salt-induced increase in ABA concentration. Sibole et al. (1998) found that increased leaf ABA during salinity stress is directly associated with leaf Na^+^ concentration. Moreover, ABA content decreased in response to salt stress under AuNPs application, possibly attributed to accumulation of K^+^ ions.

An increase in stress biomarkers (H_2_O_2_, O_2_^· −^, MDA, LOX and EL) in plants exposed to salinity is one of the most often seen phenomena (Du et al., 2015; Morales and Munné-Bosch, 2019). It becomes clear that excessive Na^+^ accumulation in the cytoplasm has been found to negatively impact the uptake of other ions (K and Ca), which in turn affects the nitrogen assimilation (Debouba et al., 2006) and AsA-GSH cycle (Hasanuzzaman et al., 2018). Furthermore, many enzymes rely on K ions for catalytic function; Na ions, on the other hand, eventually cause nutritional imbalances (Yang and Guo, 2018) and increased ROS generation causes oxidative stress and an increase in LOX, prompting lipid peroxidation (Kong et al., 2016) indicated by an increase in MDA and electrolyte leakage respectively. Additionally, ROS accumulation beyond a threshold leads to the oxidation of proteins and nucleic acids, contributing to cellular dysfunction and structural malfunction. Therefore, the regulation of LOX, MDA and ROS levels through an activated defense system is an essential mechanism for inducing stress tolerance in plants. An effective deterioration in these stress biomarkers was observed under S-AuNPs supplemented spinach in contradiction with NaCl treatment (**Figures 5**, **6**). This could be attributed to the activation of ROS scavenging systems, which modulate the defense system by activating the ascorbate-glutathione pathway (Noctor and Foyer, 1998). This pathway includes non-enzymatic antioxidants such as AsA and GSH, as well as enzymatic antioxidants like SOD, CAT, APX, POD, GPX and GR. The active involvement of these antioxidants under S-AuNPs application resulted in reduced ROS accumulation, inhibition of lipid peroxidation, and modulation of K^+^ efflux by deactivating hydroxyl radical-activated K^+^ channels (Demidchik et al., 2010).

Moreover, the increase in superoxide dismutase (SOD) activity following S-AuNPs application (**Figure 8A**) reveals how plants respond to salt stress and how S-AuNPs aid in stress mitigation. SOD is vital for scavenging superoxide radicals during stress, preventing oxidative damage to cellular components. This up-regulation of SOD activity underscores the importance of antioxidant defenses against salt-induced oxidative stress. S-AuNPs likely enhance this defense mechanism, reducing oxidative damage and mitigating salt stress effects. By converting superoxide radicals into hydrogen peroxide, SOD contributes to ROS detoxification, further maintaining cellular homeostasis (Cavalcanti et al., 2004) and works in alignment with CAT and APX (**Figures 8B, C**) which decomposes H_2_O_2_ into water and oxygen (Kumar et al., 2013). In addition, increased POD (**Figure 8D**) activity under AuNPs is due to its active involvement ROS scavenging in salt stress plants (Gunjan et al., 2014). According to Wahid et al. (2022), the ability of AuNPs to stimulate endogenous nitric oxide synthesis, which prompts the expression of many ROS scavenging enzyme genes in plants is responsible for this increase in antioxidant enzyme levels (Manaf et al., 2021; Zia-ur-Rehman et al., 2023). Similar results under application of AuNPs were also demonstrated in lavender (Jadczak et al., 2020) and spinach (Amir et al., 2024). Additionally, the plants exposed to severe salt stress showed an elevation of GR and GPX activities (**Figures 8E, F**), but this was insufficient to maintain a reduced glutathione pool and limited glutathione production, which led to severe oxidative damage (I. Khan et al., 2020; Wahid et al., 2022). Alternatively, S-AuNPs increases in AsA and GSH content (**Figures 7E, F**) suggest beneficial interaction between AuNPs and non-enzymatic antioxidants in the presence of salt stress. Furthermore, these mentioned antioxidants contribute to the maintenance of redox homeostasis in the spinach plants treated with S-AuNP.

In addition to ROS scavenging enzymes, plants also generate various secondary metabolites like proline and flavonoids, which enhance tolerance to salinity. The synthesis and accumulation of these metabolites under salinity plays significant role in membrane protection, biomolecules stability and maintenance of normal cell function (Hayat et al., 2012; Per et al., 2017). In this study, S-AuNPs supplementation significantly improves the proline and flavonoid contents (**Figures 5F**, **7D**); aiding in osmotic adjustment and safeguarding sub-cellular structures under salt stress. Similar results under salinity by NPs application were reported in tomato (Faizan et al., 2021), pearl millet (Khan et al., 2020) and wheat (Zafar et al., 2024).

Essential nutrients are crucial for the healthy growth of plants. Several studies have demonstrated that salinity limits the uptake and movement of essential minerals in plants (Handa et al., 2018; Ahmad et al., 2019; Zaheer et al., 2020). Similarly, in our study Na^+^ content was increased under NaCl irrigation and competitively inhibits the uptake of other cations, including K^+^, Ca^2+^, Fe^2+^, Mn^2+^ and Zn^2+^ in shoot of plants (**Table 2**); resulting in oxidative stress, disruption of Ca^++^ and K^+^ functions, and an imbalance in cellular homeostasis. Specifically, the imbalance in Na^+^/K^+^ and Na^+^/Ca^++^ ratios alters physiological characteristics of plants, affecting photosynthesis and plant growth. Our finding suggest that, application of AuNPs regulates the ionic ratio of K^+^/Na^+^, leading to decreased Na^+^ accumulation and increased K^+^ levels and other minerals in spinach plants. This adjustment positively affects stomatal traits, led to enhanced gas exchanges and photosynthates production, and results in improved plant growth under salt stress.

## 5 Conclusion

In conclusion, the utilization of phytofabricated AuNPs has demonstrated considerable potential in eliciting favorable physiological and biochemical responses in plants, particularly in mitigating salt stress. Our findings suggest that soil irrigation with S-AuNPs effectively alleviated salt-induced adversities in spinach, leading to positive impacts on various growth attributes, chlorophyll content, stomatal traits, K^+^ ionic concentration, ABA production, stress marker accumulation, antioxidant activity, as well as metabolites and nutritional content. The modulation of these traits by S-AuNPs underscores their potential role in inducing salt stress tolerance in plants, highlighting the importance of employing greener approaches for NPs production in agricultural practices aimed at enhancing crop resilience and productivity under adverse environmental conditions. However, further research is warranted to delve into AuNPs-induced transcriptomic changes under various stress conditions. This will contribute to a deeper understanding of the traits influencing gene interactions in plant responses to NPs, thus paving the way for more targeted approaches in enhancing plant stress tolerance.

## Data availability statement

The raw data supporting the conclusions of this article will be made available by the authors, without undue reservation.

## Author contributions

MA: Conceptualization, Data curation, Investigation, Methodology, Software, Visualization, Writing – original draft, Writing – review & editing. AR: Data curation, Methodology, Writing – review & editing. PY: Methodology, Writing – review & editing. VK: Methodology, Writing – review & editing. RKT: Methodology, Writing – review & editing. SUJ: Formal analysis, Writing – review & editing. MD: Methodology, Writing – review & editing. MIA: Funding acquisition, Resources, Supervision, Validation, Writing – review & editing.
